# Some Phases of Dental Education

**Published:** 1904-09

**Authors:** A. E. Baldwin

**Affiliations:** Chicago


### SOME PHASES OF DENTAL EDUCATION.[1]

BY A. E. BALDWIN, M.D., D.D.S., LL.B., CHICAGO.

In order to realize that we are living at a very rapid rate, we have but to look backward to see the progress that has beeen made in almost all lines within the memory of even the younger men of the profession. By observation we are shown that the educa-

[1] Read at the fifty-fifth annual session of the American Medical Association, in the Section on Stomatology, Atlantic City, June 7 to 10, 1904.

tion required to enable one to call one's self an educated artisan, scientist, or professional person was very meagre as compared with the demands and the requirements of to-day.

The question often arises in my mind whether or not stomatology, as a specialty, has kept pace with the march of progress in other lines, and especially in coincident professional lines. Much has been written, much is being written, and much more is being thought, as to the proper lines of progressional development in education in our specialty. Within the past few years we have seen that the National Association of Dental Faculties has, after long discussions, extended the requirements, preliminary and graduating, until now the time required in our most progressive schools is nothing less than a four years' course, with more strict preliminary requirements. However, by noticing the actions of some of our educators, we fear that there is a movement on foot—especially in the schools which are conducted by private ownership—because of a fear that with these added requirements the collegiate expenses will be increased, with a corresponding decrease in receipts; consequently these schools—which are conducted, if you please, for financial gains incidentally—look on the matter as a strictly mercantile transaction, and I fear that there may be a retrogressive move lessening the preliminary requirements, and also lessening the amount of time necessary to be spent in schools in order to graduate.

It has been my observation for a score of years that there are many people practising with us who seem to have a fear of acquiring too broad and comprehensive an education; indeed, a short time ago a paper was read before the Odontological Society of Chicago by Dr. H. T. Carlton, San Francisco, in which he advocated a radical advance in not only the preliminary requirements for entering our colleges, but also an emphatic advance in the requirements which must be met before graduation is allowed. This paper was read before the largest assemblage of dentists ever gathered in the world, and, much to my surprise, many men who had been classed with those who were in favor of advanced education showed by their discussion of this paper that apparently they were afraid that the young men entering our field might be too broadly educated and too well grounded in knowledge, not only of the whole human frame, but in all other scientific directions. It was convincing to a reasoning and observing mind that the only hope for rapid and thorough educational progress in our field lies in the schools which are maintained and conducted otherwise than for financial ends. In other words, the hope of dentistry, in its progressive advance, lies more largely than we will admit, first, in the schools which are founded and conducted in connection with our great colleges and universities, and, second, in the gradual elimination of the schools which are conducted by organized capital for the income which it produces, or by the organized professional band who, for notoriety or gain, organize and conduct schools which apparently are more anxious for numbers than for intellectual prominence. This is said with regret and with no intention to belittle or attack any school or college in particular, but with recognition of the fact that if the teachers or the organization have a direct financial interest in the income, gross and net, it must hamper or modify the influence which the school and teachers may exert over the student and the requirements demanded of him.

A great movement in advance can be forced if our national organization of the several State boards unify and exact constantly advancing requirements for entering our chosen field.

